# Biofilm Formation by Uropathogenic *Escherichia coli* Is Favored under Oxygen Conditions That Mimic the Bladder Environment

**DOI:** 10.3390/ijms18102077

**Published:** 2017-09-30

**Authors:** Allison R. Eberly, Kyle A. Floyd, Connor J. Beebout, Spencer J. Colling, Madison J. Fitzgerald, Charles W. Stratton, Jonathan E. Schmitz, Maria Hadjifrangiskou

**Affiliations:** 1 Department of Pathology, Microbiology and Immunology, Vanderbilt University Medical Center, Nashville, TN 37232, USA; allison.r.eberly@vanderbilt.edu (A.R.E.); kafloyd@ucsc.edu (K.A.F.); connor.j.beebout@vanderbilt.edu (C.J.B.); spencer.colling@outlook.com (S.J.C.); charles.stratton@vanderbilt.edu (C.W.S.); jonathan.e.schmitz@Vanderbilt.Edu (J.E.S.); 2 Department of Biological Sciences, Vanderbilt University, Nashville, TN 37235, USA; madison.fitzgerald@vanderbilt.edu; 3 Department of Medicine, Vanderbilt University Medical Center, Nashville, TN 37232, USA; 4 Department of Urologic Surgery, Vanderbilt University Medical Center, Nashville, TN 37232, USA

**Keywords:** bacterial biofilms, *E. coli* respiration, terminal electron acceptor, urinary tract infection, uropathogenic *E. coli*

## Abstract

One of the most common urologic problems afflicting millions of people worldwide is urinary tract infection (UTI). The severity of UTIs ranges from asymptomatic bacteriuria to acute cystitis, and in severe cases, pyelonephritis and urosepsis. The primary cause of UTIs is uropathogenic *Escherichia coli* (UPEC), for which current antibiotic therapies often fail. UPEC forms multicellular communities known as biofilms on urinary catheters, as well as on and within bladder epithelial cells. Biofilm formation protects UPEC from environmental conditions, antimicrobial therapy, and the host immune system. Previous studies have investigated UPEC biofilm formation in aerobic conditions (21% oxygen); however, urine oxygen tension is reduced (4–6%), and urine contains molecules that can be used by UPEC as alternative terminal electron acceptors (ATEAs) for respiration. This study was designed to determine whether these different terminal electron acceptors utilized by *E. coli* influence biofilm formation. A panel of 50 urine-associated *E. coli* isolates was tested for the ability to form biofilm under anaerobic conditions and in the presence of ATEAs. Biofilm production was reduced under all tested sub-atmospheric levels of oxygen, with the notable exception of 4% oxygen, the reported concentration of oxygen within the bladder.

## 1. Introduction

### 1.1. Urinary Tract Infection in the Context of Benign Urologic Disease

Urinary tract infections (UTIs) account for 20 million hospital or clinic visits and billions of dollars in healthcare expenditures annually in the United States and Europe [1,2,3,4]. The most common type of UTI is acute cystitis, which is characterized by urinary urgency, frequency, dysuria, and pyuria [4,5]. UTIs primarily afflict women, with one in two experiencing at least one UTI episode in her lifetime [4]. Approximately 30% of women with a UTI will have recurrent infection [4,6]. Men are less susceptible to UTIs than women, but their risk of infection increases with age. Furthermore, infected men are more likely to experience more severe forms of infection, such as pyelonephritis and urosepsis [7,8]. UTIs are a common complication among hospitalized patients, particularly among the elderly and patients with diabetes, bladder cancer, or indwelling catheters [9,10]. Current therapy for UTIs often fails, and this is exacerbated by the increased incidence of UTIs caused by bacteria resistant to common antimicrobials [11,12].

### 1.2. Uropathogenic Escherichia coli Biofilm Formation

Uropathogenic *Escherichia coli* (UPEC) is the most common cause of UTI, accounting for approximately 80% of infections [2]. UPEC readily forms multi-cellular communities known as biofilms on the surface of catheter materials, bladder walls, as well as within bladder epithelial cells [13,14,15,16]. The formation of biofilms markedly impedes the treatment of UTIs by protecting encased bacteria from both the host immune response and antimicrobial therapy. Bacteria are in close proximity within the biofilm, facilitating the exchange of genetic material, such as antimicrobial resistance plasmids and transposons [17,18]. Understanding the host factors that facilitate biofilm formation during UTIs will enhance the development of better strategies for combatting biofilms and treating infections.

To form a biofilm, bacteria must first attach to a surface. Adherence of UPEC strains can be influenced by a wide variety of intrinsic factors, such as adhesive proteins, fibers, and exopolysaccharide molecules; the carriage and expression of such factors differs from strain to strain [19,20,21,22,23,24]. Once attached to the surface bacteria change from a planktonic form to a sessile form, which replicate while producing extracellular matrix (ECM). This ECM encases the bacteria in a micro-colony. The ECM can be made up of one or more components including exopolysaccharides, proteinaceous material, and extracellular DNA [17,25,26,27,28]. As the biofilm colony grows and matures, bacteria within the ECM respond to signals from their surrounding environment, eventually leading to a portion of the encased bacteria dispersing from the biofilm colony [19,29,30]. The dispersed bacteria can return to the planktonic form or continue the process of biofilm formation elsewhere. Alternatively, the bacteria can form quiescent reservoirs in the urothelium which are thought to contribute to recurrent infection [31,32,33].

**Figure 1:**
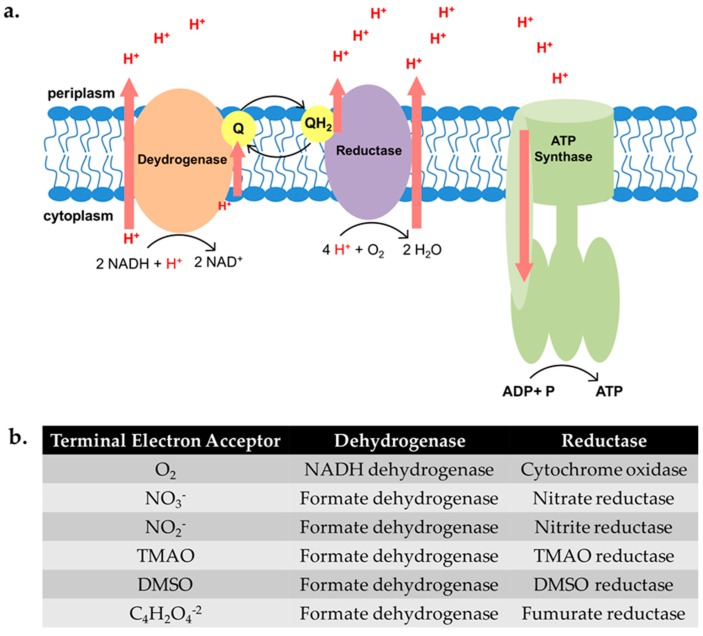
Schematic depicting critical enzymes in the electron transport chain. (**a**) The schematic represents the enzymes utilized to generate energy when oxygen is the terminal electron acceptor. Aerobic respiration yields the most ATP, undergoing glycolysis and the tricarboxylic acid (TCA) cycle to feed NADH molecules to the electron transport chain (ETC), where the bulk of ATP is produced. In the case of aerobic respiration when oxygen is abundant, the electron transport chain, which is located in the inner membrane, consists of an NADH dehydrogenase that donates electrons to the cytochrome *bo* oxidase. The transfer of electrons between the enzymes is mediated by ubiquinones (Q, QH_2_). The reduction of oxygen to water by the cytochrome oxidase creates a proton motive force across the membrane, and these protons are used to drive ATP synthase to produce ATP. In the absence of oxygen and in the presence of an alternative terminal electron acceptor, *E. coli* undergo anaerobic respiration. *E. coli* encodes two NADH and at least two formate dehydrogenases that are utilized under different respiration conditions; (**b**) the table shows the dehydrogenase and reductase enzymes used for each terminal electron acceptor. *E. coli* is a facultative anaerobe, having the ability to use terminal electron acceptors other than oxygen, or generating energy via mixed acid fermentation. *E. coli* can utilize five additional terminal electron acceptors in the absence of oxygen (O_2_) in the following preferential order: nitrate (NO_3_^−^), nitrite (NO_2_^−^), dimethyl sulfoxide (DMSO), trimethylamine-*N*-oxide (TMAO), and fumarate (‎C_4_H_2_O_4_^−2^).

UPEC is a facultative anaerobe with significant metabolic versatility. Despite this, previous work has shown that deletion of genes encoding the tricarboxylic acid (TCA) cycle enzymes attenuates UPEC infection in a well-characterized murine model [34,35,36]. The TCA cycle generates molecules, such as NADH and FADH, that can be utilized in the electron transport chain if oxygen or one of five alternative terminal electron acceptors (ATEAs) is available (Figure 1 and [37,38]). In addition, our group has demonstrated that UPEC mutants unable to use oxygen as the terminal electron acceptor are also attenuated for virulence [39]. Together, these findings suggest that UPEC isolates use aerobic respiration during UTIs.

In this study, we sought to evaluate the amount of biofilm produced when wild UPEC strains are grown in anaerobic conditions in the presence of ATEAs. We demonstrate that UPEC biofilm formation is decreased under anoxic conditions compared to atmospheric conditions in 50 urine-associated *E. coli* clinical isolates. Furthermore, addition of ATEAs to the growth medium did not restore biofilm formation for any of the strains tested. Evaluating biofilm formation in decreasing concentrations of oxygen revealed that only hypoxic conditions that mimic the oxygen concentration of the bladder (4–5.5% oxygen; [40]) led to robust biofilm formation.

## 2. Results

### 2.1. Uropathogenic Escherichia coli (UPEC) Biofilm Formation Is Diminished under Anoxic Conditions

**Figure 2:**
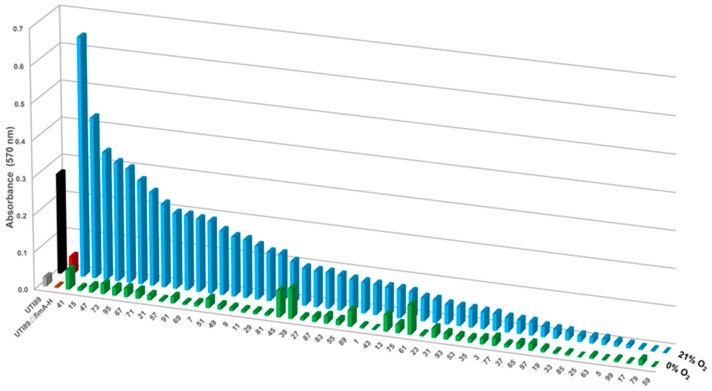
The majority of urine-associated *E. coli* clinical isolates exhibit decreased biofilm formation under anoxic conditions independent of type 1 pili production. Graph depicting the quantified biomass for 50 urine-associated *E. coli* strains collected from the urine of patients at VUMC. These randomly-selected isolates were seeded in standard biofilm plates and allowed to develop biofilms along the wall of the wells for 48 h in ambient oxygen conditions. The Z-axis depicts the oxygen concentration under which the isolates were grown. Urine-associated *E. coli* isolates are graphed from the highest biofilm production to lowest under 21% oxygen (blue bars). UTI89 (black bars) and the isogenic UTI89*∆fimA-H* mutant (red bars) were used as controls for comparison of the assay. All isolates exhibited significantly-reduced biofilm compared to their own growth under ambient oxygen conditions, except isolates VUTI39 and VUTI61 (green bars). The average of a minimum of two independent experiments is shown, with a minimum of eight technical replicates per experiment. The standard error of the mean for each VUTI isolate is shown in Table S2.

**Figure 3:**
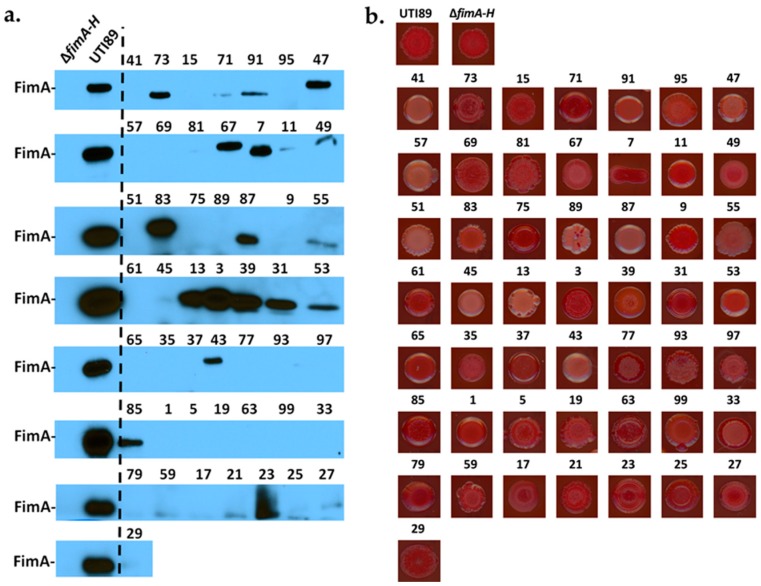
Urinary isolates form variable levels of known biofilm matrix components. (**a**) Immunoblot depicts the abundance of FimA pilin subunit in the 50 VUTI strains. Cultures were grown statically overnight under atmospheric conditions. The antibody used to probe for FimA production was raised against the FimA antigen of cystitis strain UTI89 [45], which is used here as a positive control. The size of UTI89 FimA is roughly 18.5 kDa. An isogenic strain deleted for the entire *fim* operon (*∆fimA-H*) is used as a negative control. This immunoblot indicates that 27/50 isolates express a FimA isoform or a pilin subunit recognized by the anti-FimA antibody used. The immunoblot shown is representative of a minimum of two biological replicates; (**b**) VUTIs were spotted on yeast extract/casamino acids (YESCA)–Congo Red agar and grown at room temperature for seven days. Congo Red uptake serves as a proxy to cellulose and curli presence in the extracellular matrix. Representative images of two independent experiments are shown here.

One of the most critical adherence factors identified in UPEC is a class of fibers termed type 1 pili [41]. Type 1 pili mediate UPEC adhesion to bladder epithelial cells, and their deletion greatly diminishes biofilm formation under laboratory and in vivo conditions [42]. We have previously shown that in the complete absence of oxygen, expression of type 1 pili is reduced in cystitis strain UTI89 [43]; mutants unable to aerobically respire also display a defect in type 1 pili production and biofilm formation [39]. These observations have led to the hypothesis that anoxic conditions reduce biofilm formation due to the reduction of type 1 pili. We, therefore, compared in vitro biofilm formation at atmospheric (21% oxygen) and anoxic conditions (0% oxygen) for a panel of urine-associated *E. coli* isolates obtained from the Vanderbilt University Medical Center (VUMC) clinical microbiology laboratory (IRB #151465). Even though the assay has not been done on bladder tissue, numerous studies have used this method for evaluating biofilm formation in vitro [39,44] As expected, cystitis isolate UTI89 exhibited decreased biofilm formation under anoxic conditions (Figure S1A), presumably due to reduced expression of these adhesive fibers [43]. This reduction in biofilm formation did not appear to be a function of reduced bacterial cell viability (Figure S1B,C and Table S1). We then quantified the biofilm levels formed by 50 urine-associated *E. coli* strains during growth in 21% versus 0% oxygen conditions in vitro (Figure 2). Biofilm levels varied among the urine-associated *E. coli* isolates, with Vanderbilt Urinary Tract Isolate 41 (VUTI41) forming the highest levels of biofilm (Figure 2 and Table S2). A subset of VUTIs was grown under atmospheric oxygen conditions and exhibited growth comparable to UTI89 (Figure S2). Interestingly, VUTIs also expressed variable levels of type 1 pili (as indicated by the abundance of the main pilin subunit FimA (Immunoblots, Figure 3a), but this expression did not correspond to levels of biofilm formation (Figure 3a). The *E. coli* matrix typically comprises curli, cellulose, colonic acid, and other adhesive moieties, depending on the strain background. To gain an appreciation in the variation of curli and cellulose produced by the 50 VUTIs, the strains were spotted on yeast extract/casamino acid (YESCA) agar supplemented with Congo Red. Congo Red uptake (indicative of cellulose and curli production) was also variable in the VUTIIs, (Figure 3b), indicating high variability in the matrix composition and possibly architecture among different isolates. Irrespective of type 1 pili expression and curli/cellulose production, 48/50 urine-associated *E. coli* isolates formed less biofilm under anaerobic conditions (Figure 2), suggesting that oxygen regulates additional biofilm factors other than type 1 pili. To more closely mimic the bladder environment, a subset of urine-associated *E. coli* isolates was tested for in vitro biofilm levels when grown in artificial urine. The biofilm levels formed by these strains in artificial urine was higher overall by crystal violet staining compared to the levels of biofilm formed when grown in LB, but greatly diminished in the absence of oxygen (Figure S3).

### 2.2. Biofilm Formation in UPEC Is Not Restored in the Presence of ATEAs

**Figure 4:**
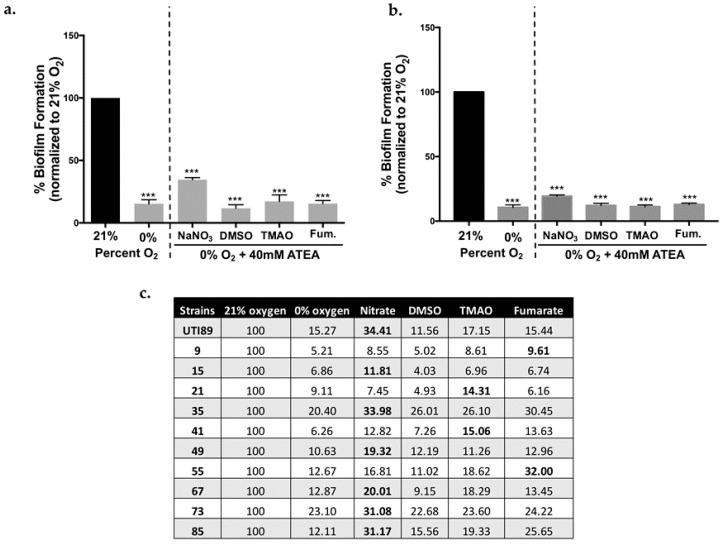
Alternative terminal electron acceptors do not restore biofilm formation. (**a**) Graph showing percent biofilm formation normalized to a well-characterized cystitis isolate UTI89 grown in the presence of oxygen (21%). UTI89 biofilm formation at 21% oxygen is artificially set to 100%, while the absorbance of the biofilm formed at 0% oxygen as well as at 0% oxygen with other terminal electron acceptors (40 mM) is averaged and then divided by the 21% oxygen average to calculate the percentage. Error bars represent the standard error of the mean (SEM) compared to UTI89 grown under 21% oxygen, *** *p*-value > 0.001; (**b**) Clinical isolate VUTI49 was tested under 21% oxygen and 0% oxygen with 40 mM of each alternative terminal electron acceptor. Percentages were calculated as in panel A and SEM is calculated compared to VUTI49 grown at 21% oxygen, *** *p*-value > 0.001; (**c**) Table including the percentages of biofilm formed by UTI89 and 10 randomly selected urine-associated *E. coli* from the panel tested in Figure 2. The highest percent biofilm formed by each strain is bolded to show the ATEA that had most effect on biofilm. The percentages were calculated as described above. Experiments were performed a minimum of three independent times.

*E. coli* can use different (i.e., alternative) terminal electron acceptors when oxygen is unavailable (Figure 1). Depending on a person’s diet, urine can be rich in trimethylamine-*N*-oxide (TMAO) [46] and nitrate [47]. Moreover, high levels of nitrite are suggestive of a UTI, as members of the Enterobacteriaceae will reduce nitrate to nitrite [47]. Dimethyl sulfoxide (DMSO) has been used for the intravesical treatment of cystitis [48,49]. It is, therefore, possible that biofilm formation may be restored under anaerobic conditions provided an ATEA is present. Our previous work has demonstrated that the addition of nitrate partially restored type 1 pili expression under anaerobic conditions [43]. We, therefore, tested biofilm formation under anaerobic conditions using 40 mM of an ATEA (Figure 4 and Figure S4). Under these conditions, cystitis strain UTI89 did not form an appreciable biofilm (Figure 4a). The presence of an ATEA also failed to restore the biofilm formation in ten randomly-selected urine-associated *E. coli* isolates (Figure 4b,c), indicating that our observations were not strain-specific.

### 2.3. Oxygen Concentrations That Mimic the Bladder Support Robust Biofilm Formation

**Figure 5:**
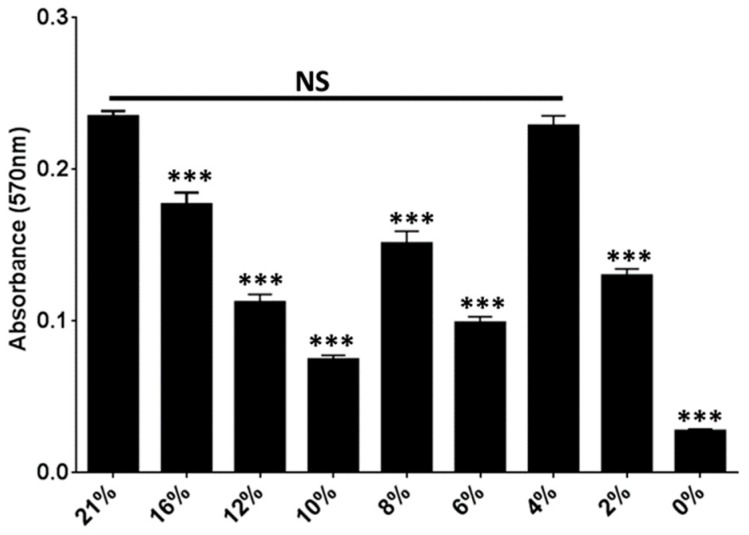
UTI89 biofilm formation is affected by oxygen concentration changes. Graph depicting the relative abundance of biofilm formed by UTI89 during growth in decreasing oxygen concentrations. This graph depicts the average of a minimum of three biological replicates per condition tested. Statistical analysis was performed using two-tailed Mann-Whitney method, comparing values for each condition to the values of biofilm formed under atmospheric (21%) oxygen. *** *p* < 0.0001, NS not significant.

The urinary oxygen tension ranges from 4–5.5% in the bladder of healthy individuals [40]. In the gut, oxygen concentration ranges from 8% oxygen in the upper GI tract to 0% in the sigmoid colon [50]. We, therefore, quantified biofilm formation at oxygen concentrations that are likely to be encountered by *E. coli* in the host. These experiments revealed that biofilm formation decreased in a step-wise fashion from 21% to 10% oxygen (Figure 5). However, biofilm levels fluctuated in a cyclical fashion as oxygen decreased from 8% to 2% oxygen, with biofilm levels at 4% oxygen reaching those obtained during growth under atmospheric oxygen conditions (Figure 5).

## 3. Discussion

In this investigation, we have demonstrated that oxygen is the terminal electron acceptor that supports the most robust biofilm production by UPEC. Though biofilm production under ambient oxygen conditions varied among isolates (Figure 2), the decrease in biofilm abundance in the absence of oxygen was consistent in 96% of all urine-associated *E. coli* tested, suggesting that the requirement for oxygen is a broad phenomenon. Notably, of the 50 clinical *E. coli* isolates tested, VUTI39 and VUTI61 formed low levels of biofilm that did not change in response to the presence or absence of oxygen. It is important to note that there are few studies that indicate whether higher levels of biofilm production correlate with more virulent *E. coli* strains, or *E. coli* strains that cause catheter-associated UTIs [51]. However, the observation that oxygen enhances the production of biofilm suggests that UPEC are exposed to ideal conditions for biofilm formation in the bladder. Furthermore, biofilm formation by UPEC exhibits a cyclical pattern from 10% to 0% oxygen. Ongoing studies are investigating if cytochrome *bd* and/or *bd*_2_ oxidase are contributing to the biofilm levels under different levels of hypoxia.

Under ambient oxygen concentrations, we have observed a wide variability in biofilm production by urine-associated *E. coli* strains (Figure 2). Colorimetric assays, such as the one used in our studies, serve as an indicator of biofilm production [52]. Biofilm production can vary as a function of bacterial numbers within the biofilm colony, as well as the amount and type of ECM produced by a given strain. For example, the high biofilm levels of strain VUTI41 could be due to higher numbers of bacterial cells within the biofilm and/or due to higher levels of ECM produced by that particular strain.

Adherence is a critical step that is required for biofilm formation by *E. coli*. Adherence to a surface can vary as a function of the expression, abundance, and type of adhesive fibers expressed by each *E. coli* strain. Type 1 pili appear to mediate surface attachment and, thus, contribute significantly to UTIs caused by UPEC [41]. We have previously reported that type 1 pili expression is decreased during UPEC growth in the absence of oxygen, and that type 1 pili are localized to the air-exposed region of biofilms formed by strain UTI89 in ambient oxygen conditions [43]. Though the majority of *E. coli* strains harbor the genes encoding for type 1 pili, not all urine-associated *E. coli* isolates expressed type 1 pili under the conditions tested. Of the 50 isolates tested, only 27 isolates express FimA under the growth conditions tested (Figure 3a). Notably, there is variation in the FimA-reactive bands in the immunoblots, indicative of the presence of different FimA isoforms, as in the case of UPEC strain CFT073 FimA (Figure S5 and [22,53]). It is, however, also possible, and we acknowledge, that there could be cross-interaction with a pilus subunit from another chaperone-usher pathway system. Most notably, there was no correlation between type 1 pili expression and biofilm abundance, as exemplified by VUTI41, which formed the most biofilm but did not produce type 1 pili (Figure 2 and Figure 3a). This strongly suggests that type 1 pili are only partially responsible for the loss of biofilm production in the absence of oxygen. Previous studies have established that the regulation of adhesive fibers is inter-connected [36,54]. It is possible that the urine-associated *E. coli* strains that do not express type 1 pili possess other functional adhesins on their cell surfaces that allow adherence. Interestingly, based on the congo-red profiles, it appears that curli and cellulose levels also vary among the clinical isolates tested (Figure 3b). VUTI41 does not uptake Congo red, suggesting that its high levels of biofilm are not attributed to type 1 pili, cellulose, or curli. Ongoing studies are aimed at determining the oxygen-mediated regulators of biofilm production in UPEC.

## 4. Materials and Methods

### 4.1. Bacterial Strains

Cystitis isolate UTI89 [33] and previously-constructed mutant *UTI89∆fimA-H* (a gift from Scott Hultgren) were used as reference strains. Urine-associated *E. coli* strains VUTI1–VUTI112 (VUTI, Vanderbilt Urinary Tract Isolate) were isolated from positive-culture urines (mono-species culture) by the Vanderbilt University Medical Center (VUMC) clinical microbiology laboratory (Nashville, Tennessee), under IRB #151465. Of the collected isolates, 50 strains were randomly selected for the biofilm studies presented in this manuscript.

### 4.2. Biofilm Assays

All strains were grown statically overnight at 37 °C in lysogeny broth (LB). Cultures were then diluted to an OD_600_ of 0.06 in fresh LB or artificial urine and seeded into 96-well polyvinyl chloride (PVC) plates as previously described [39]. Artificial urine recipe can be found in the supplementary material. Plates were incubated statically at room temperature as previously described [39], but with varying oxygen concentrations: (a) aerobic (ambient/21%); (b) hypoxic gradient; or (c) anoxic (0% oxygen) conditions. Biofilm plates grown under anoxic conditions were incubated in a vinyl anaerobic chamber (Coy Lab Products, Grass Lake, MI, USA) maintained at 0% oxygen and 2–3% hydrogen. Hypoxic oxygen concentrations were achieved in a Herthem incubator (ThermoFisher, Waltham, MA, USA) equipped with a ProOx 110 compact oxygen controller (BioSpherix, Parish, NY, USA) that displaces oxygen with nitrogen gas. All biofilm plates were incubated for 48 h at room temperature prior to quantitation. Biofilm abundance was quantified using the crystal violet staining method of O’Toole et al. [52]. For the ATEA biofilm experiments, the following procedure was performed in addition to what is described above: A 3.2 M stock concentration of nitrate, DMSO, or TMAO was diluted with sterile water to the desired final concentration in each well (ranging from 160 to 20 mM) so that the final liquid volume in each well was equal. Fumarate was diluted in LB to a stock concentration of 160 mM and further diluted in fresh LB to achieve the desired final concentrations. Each study was performed at least three independent times, with at least 24 technical replicates per biological replicate. Congo red uptake was qualitatively assessed by spotting 5 μL of overnight culture on 1.2× yeast extract/casamino acid (YESCA) Congo red agar plates and monitoring for Congo red uptake and rugose morphology, as previously described [55,56]. Plates were incubated at room temperature and imaged on day 7. At least two independent experiments were performed.

### 4.3. Growth Curves and CFU Enumeration

Bacterial cultures were grown in LB overnight shaking at 37 °C. Overnight cultures were diluted to an OD_600_ of 0.06 in fresh LB. 100 μL of diluted culture was added to each well in a flat-bottom 96-well plate. Absorbance measurements at an optical density at 600 nm were recorded at 15-min intervals for 24 h. Growth curves under ambient oxygen conditions were performed using a SpectraMax i3 (Molecular Devices, Sunnyvale, CA, USA) plate reader, while measurements under anoxic conditions were performed using a Synery H1 hybrid reader (BioTek, Winooski, VT, USA). In addition to absorbance readings, samples were collected at 60-min intervals for CFU enumeration. Obtained samples were serially diluted and plated on LB plates (8–10 technical replicates per sample) for enumeration. Experiments were performed independently at least two times.

### 4.4. Immunoblot Analyses

All strains were grown statically overnight at 37 °C. Culture was normalized to an OD_600_ of 1 and then FimA immunoblots were performed as detailed in Floyd et al. [43].

## 5. Conclusions

This study demonstrates that oxygen is a major driver of biofilm formation across a range of UPEC isolates. Most importantly, the highest biofilm abundance is observed at ambient and at 4% oxygen concentrations. Further research is aimed at pinpointing the factors and regulatory processes that enhance biofilm formation at 4% oxygen compared to other hypoxic conditions.

## Supplementary Materials

Supplementary materials can be found at www.mdpi.com/1422-0067/18/10/2077/s1.

## Abbreviations

UTI	Urinary tract infection
UPEC	Uropathogenic *E. coli*
ATEA	Alternative terminal electron acceptor
ASB	Asymptomatic bacteriuria
CAUTI	Catheter-associated UTI
ECM	Extracellular matrix
