# Supplementary material for: Biofilm Formation by Uropathogenic Escherichia coli Is Favored under Oxygen Conditions That Mimic the Bladder Environment
Source: Int J Mol Sci. 2017 Sep 30;18(10):2077. doi: 10.3390/ijms18102077 (PMC5666759; doi:10.3390/ijms18102077)
Supplement: Supplementary file 1 [file ijms-18-02077-s001.pdf]

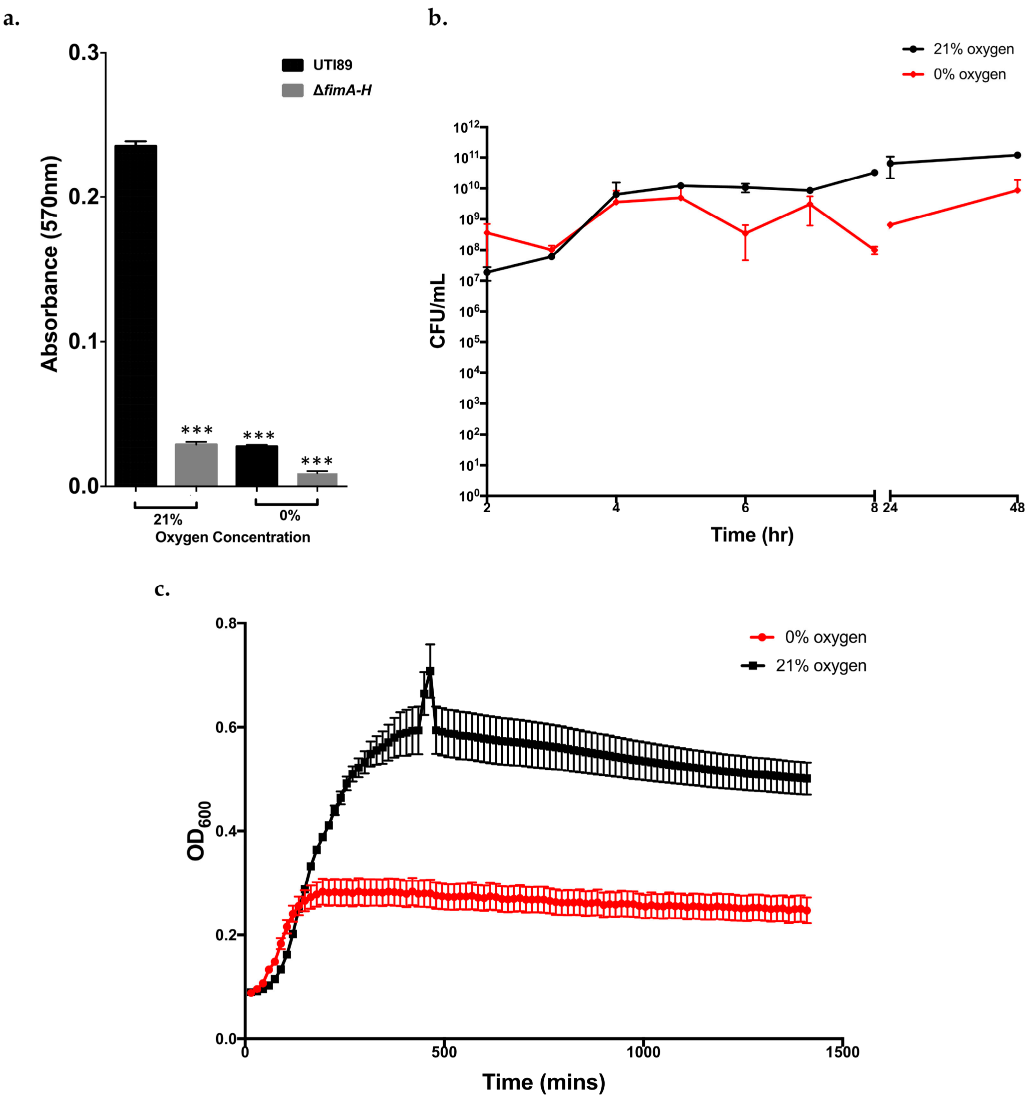

Figure S1. UPEC biofilm levels are decreased under anoxic conditions. (**a**) UTI89 biofilm formation at 0% oxygen is decreased compared to biofilm levels at 21% oxygen by 96-well crystal violet assay. UTI89*ΔfimA-H* is a negative control, as the strain lacks the ability to form type 1 pili, one of the major components of UTI89 biofilms. *** p-Value < 0.0001 by one-way ANOVA compared to UTI89 at 21% oxygen. Data shown is representative of at least 3 independent experiments with 24 technical replicates; (**b**) The decrease in biofilm formation by UTI89 is not due to a loss of cell viability. Colony forming units (CFU) show that though there is roughly a log decrease in the number of cells, the bacteria are still viable. Error bars represent standard deviation between replicates at each time point. CFU enumeration was performed at least two independent times; (**c**) Growth rate is altered during growth under anoxic conditions. Growth curves measuring the optical density at 600 nm over time show that there is not a lack of cell viability when UTI89 is grown under anoxic conditions. Error bars show standard error of the mean among technical replicates. This data is representative of at least two independent experiments, with at least 5 technical replicates per experiment.

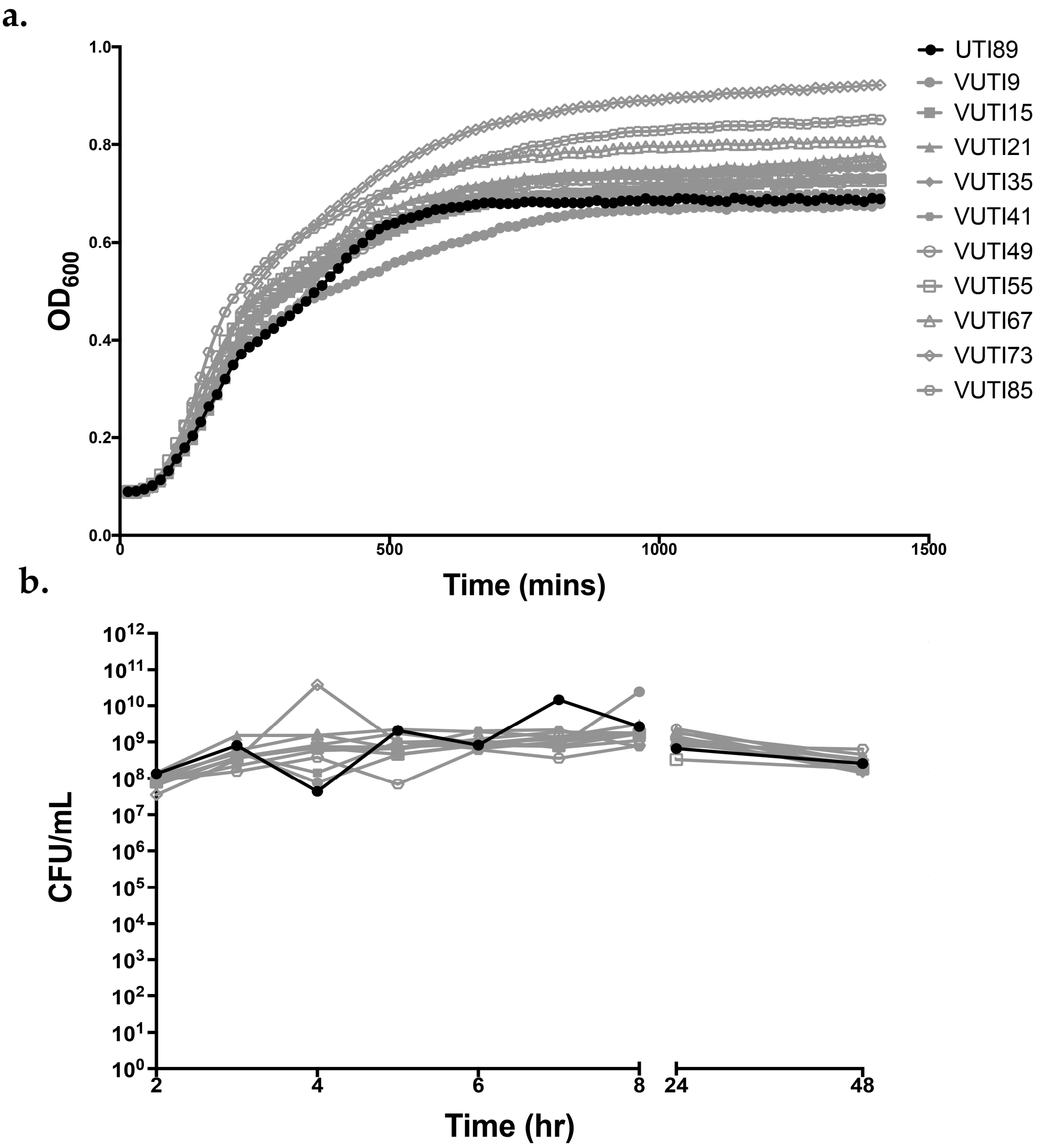

Figure S2. VUTI isolates do not exhibit growth defects compared to UTI89. (**a**) Growth rate of VUTI isolates is comparable to UTI89 under atmospheric conditions. Growth curves measuring the optical density at 600 nm over time as described in Figure S1c. This data is representative of one independent experiments, with at least 8 technical replicates per isolate; (**b**) Colony forming units (CFU) show that the bacteria are viable throughout the duration of biofilm assays performed. Assay was performed one time as described in Figure S1b.

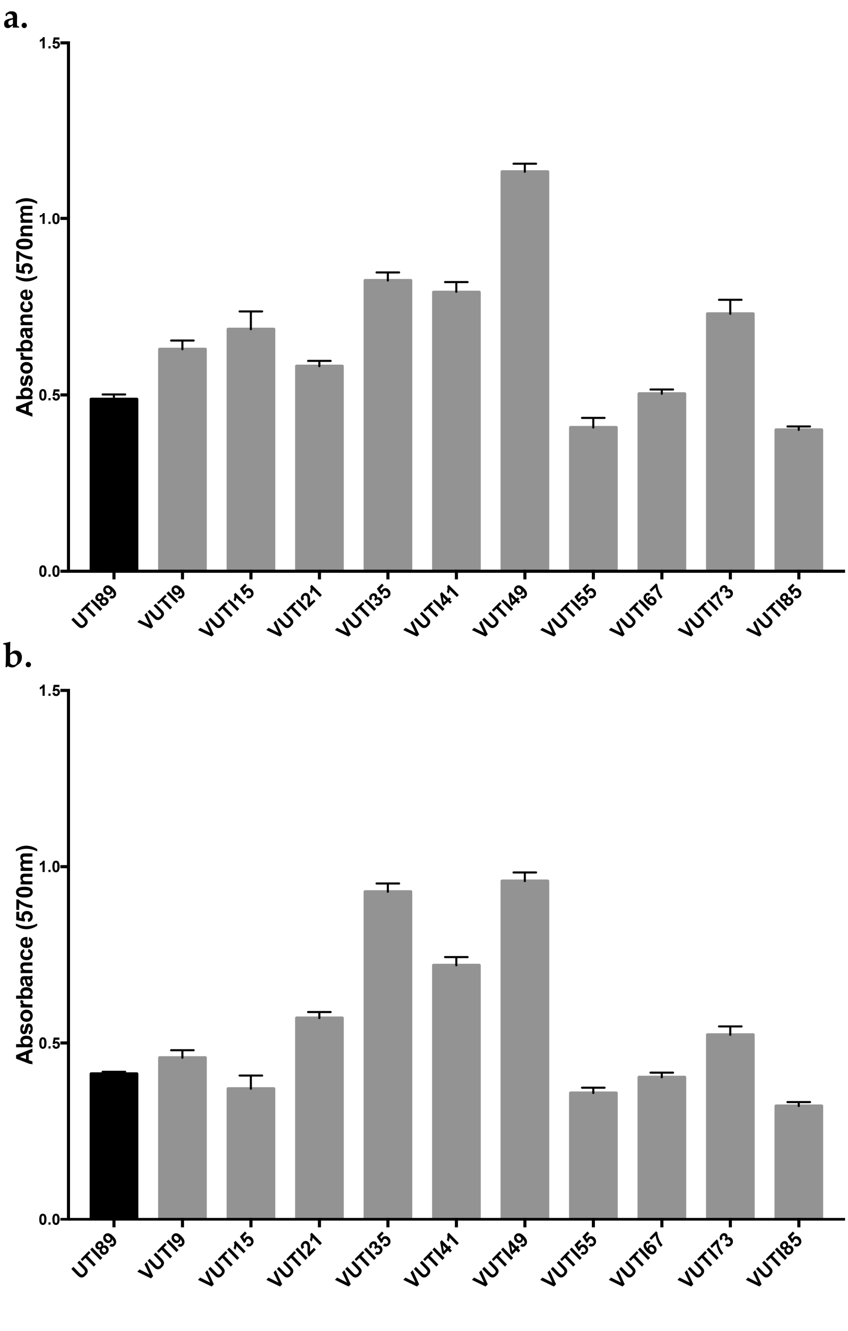

Figure S3. Biofilm formation is enhanced by artificial urine under atmospheric conditions, but not anoxic conditions. (**a**) Artifical urine increases biofilm formation among UTI89 and the VUTI isolates tested under atmospheric conditions (21% oxygen); (**b**) Biofilm formation is not restored under anoxia (0% oxygen) when isolates are grown in artificial urine. This data is representative of three biological replicates, with at least 24 technical replicates per experiment. Error bars show the standard error of the mean.

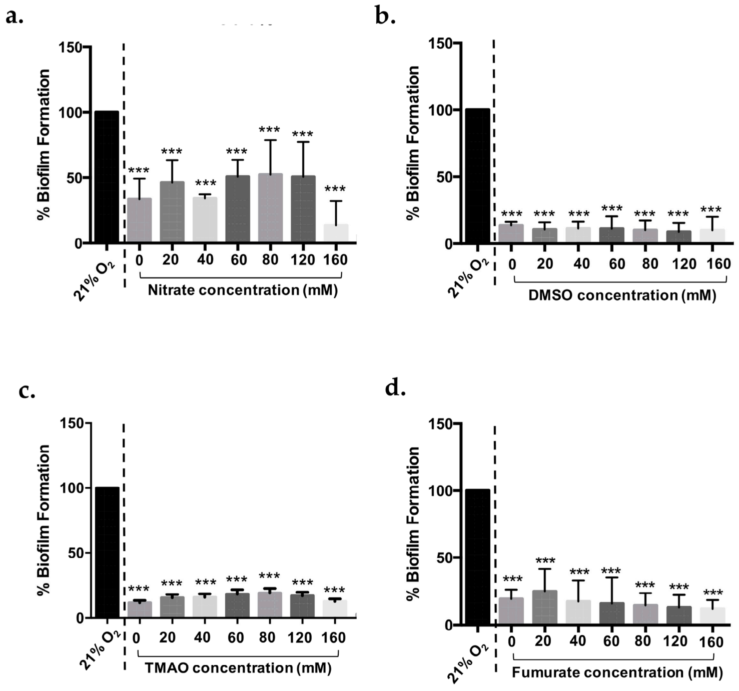

Figure S4. The concentration of alternative electron acceptors does not impact biofilm formation restoration. Each terminal electron acceptor was added in increasing concentrations from 20 mM to 160 mM. The alternative terminal electron acceptors are (**A**) nitrate, (**B**) DMSO, (**C**) TMAO, (**D**) fumarate. This data is representative of at least three independent experiments, with at least 24 technical replicates per experiment. Statistical analysis was performed with two-tailed Mann-Whitney, comparing values for each condition to the values of biofilm formed under atmospheric (21%) oxygen. *** $p < 0.0001$.

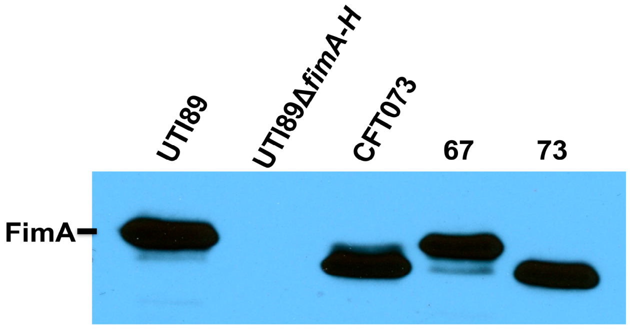

Figure S5. FimA immunoblots using normalized cultures that were grown statically overnight and used to seed biofilm assays. Isolates VUTI67 and VUTI73 express FimA as robustly as UTI89 and CFT073.

Table S1. Raw numbers from Figure S1B are represented here.

	21% oxygen		0% oxygen	
**Time (h)**	**UTI89 Rep 1**	**UTI89 Rep 2**	**UTI89 Rep 1**	**UTI89 Rep 2**
0	$2.18x10^{6}$	$1.78x10^{6}$	$5.00x10^{8}$	$4.8x10^{6}$
1	$4.80x10^{6}$	$3.20x10^{6}$	$1.04x10^{7}$	$4.2x10^{7}$
2	$2.52x10^{7}$	$1.26x10^{7}$	$1.26x10^{8}$	$6.00x10^{8}$
3	$6.00x10^{7}$	$6.00x10^{7}$	$7.40x10^{7}$	$1.28x10^{8}$
4	$2.48x10^{7}$	$1.28x10^{10}$	$7.00x10^{9}$	$1.58x10^{8}$
5	$1.32x10^{10}$	$1.12x10^{10}$	$9.80x10^{9}$	$5.00x10^{7}$
6	$8.40x10^{9}$	$1.32x10^{10}$	$1.34x10^{8}$	$5.6x10^{8}$
7	$8.40x10^{9}$	$8.80x10^{9}$	$1.34x10^{9}$	$4.8x10^{9}$
8	$3.02x10^{10}$	$3.52x10^{10}$	$1.20x10^{8}$	$8.00x10^{7}$
24	$9.52x10^{10}$	$3.38x10^{10}$	$5.80x10^{8}$	$7.20x10^{8}$
48	$1.38x10^{11}$	$1.04x10^{11}$	$1.6x10^{9}$	$1.58x10^{10}$

Table S2: Table lists the average of the absorbance at 570 nm for each of the 50 isolates screened for biofilm formation at 21% oxygen and 0% oxygen along with the standard error of the mean (SEM).

Isolate	Absorbance (570nm)	SEM	Absorbance (570nm)	SEM
UTI89	0.2701	0.00552	0.02453	0.001282
UTI89Δ*fimA-H*	0.0495	0.009396	0.00319	0.0002592
41	0.6450	0.0148	0.0531	0.001221
15	0.4328	0.012	0.0079	0.002335
47	0.3440	0.01738	0.0169	0.0002466
73	0.3201	0.04663	0.0273	0.001568
95	0.3076	0.01034	0.0219	0.0006946
67	0.2792	0.01418	0.0240	0.00157
71	0.2517	0.009984	0.0219	0.0008916
21	0.2241	0.02204	0.0138	0.002798
57	0.2033	0.01022	0.0045	0.0001542
91	0.2020	0.01758	0.0170	0.000289
69	0.1960	0.01282	0.0050	0.00081
7	0.1936	0.005538	0.0089	0.001046
51	0.1724	0.004367	0.0243	0.0007479
49	0.1601	0.01033	0.0109	0.0006613
9	0.1559	0.005457	0.0085	0.0006204
11	0.1439	0.01062	0.0063	0.001054
29	0.1309	0.01029	0.0069	0.0009281
81	0.1300	0.02071	0.0061	0.0003683
45	0.1136	0.002577	0.0718	0.0018
39	0.1000	0.006192	0.0819	0.006554
27	0.0971	0.002637	0.0078	0.00086
87	0.0968	0.01159	0.0147	0.0006153
83	0.0931	0.01722	0.0201	0.0002811
55	0.0864	0.007154	0.0136	0.0005828
89	0.0831	0.0167	0.0439	0.003411
1	0.0809	0.01481	0.0050	0.001282
43	0.0784	0.01003	0.0015	0.000119
13	0.0775	0.003347	0.0445	0.005697
75	0.0737	0.01636	0.0231	0.001676
61	0.0621	0.0106	0.0797	0.004079
23	0.0611	0.004755	0.0042	0.001644
31	0.0529	0.004267	0.0252	0.002312
93	0.0506	0.003049	0.0162	0.000741
53	0.0491	0.003226	0.0126	0.0007044
35	0.0457	0.003509	0.0114	0.00153
3	0.0447	0.007167	0.0152	0.0002592
77	0.0385	0.004075	0.0213	0.001182
37	0.0367	0.003119	0.0072	0.0005375
65	0.0356	0.006035	0.0152	0.0006716
97	0.0276	0.003931	0.0182	0.001619
19	0.0244	0.007798	0.0092	0.00109
33	0.0228	0.005141	0.0041	0.001327
85	0.0164	0.009211	0.0049	0.0004652
25	0.0157	0.004549	0.0000	0
63	0.0153	0.0009836	0.0065	0.0007633
5	0.0114	0.001413	0.0022	0.0003968
99	0.0086	0.001982	0.0055	0.0003715
17	0.0038	0.00164	0.0025	0.001025
79	0.0022	0.0004589	0.0141	0.0005627
59	0.0008	0.0003623	0.0003	0.0001268

Artificial Urine Recipe

Part 1: Dissolve sodium chloride (78.7 mM), sodium sulfate (9 mM), sodium citrate dihydrate (2.2 mM), sodium oxalate (0.1 mM), potassium dihydrogen phosphate (3.6 mM), and potassium chloride (21.5 mM) in 540 mL of water. Part 2: Dissolve 3 grams of tryptic soy broth (TSB) in 400 mL of water. Autoclave parts 1 and 2. After parts 1 and 2 cool, combine. Add solutions of calcium chloride (3 mM), magnesium chloride (2 mM), ammonium chloride (15 mM), creatinine (6 mM), and urea (200 mM). After combining, measure pH (~6.3).
